# *GRIN2A* (NR2A): a gene contributing to glutamatergic involvement in schizophrenia

**DOI:** 10.1038/s41380-023-02265-y

**Published:** 2023-09-22

**Authors:** Paul J. Harrison, David M. Bannerman

**Affiliations:** 1 Department of Psychiatry, University of Oxford, Warneford Hospital, Oxford, OX3 7JX UK; 2 Oxford Health NHS Foundation Trust, Oxford, UK; 3 Department of Experimental Psychology, University of Oxford, Oxford, OX2 6GG UK

**Keywords:** Schizophrenia, Genetics

## Abstract

Involvement of the glutamate system, particularly N-methyl-D-aspartate (NMDA) receptor hypofunction, has long been postulated to be part of the pathophysiology of schizophrenia. An important development is provided by recent data that strongly implicate *GRIN2A*, the gene encoding the NR2A (GluN2A) NMDA receptor subunit, in the aetiology of the disorder. Rare variants and common variants are both robustly associated with genetic risk for schizophrenia. Some of the rare variants are point mutations likely affecting channel function, but most are predicted to cause protein truncation and thence result, like the common variants, in reduced gene expression. We review the genomic evidence, and the findings from *Grin2a* mutant mice and other models which give clues as to the likely phenotypic impacts of *GRIN2A* genetic variation. We suggest that one consequence of NR2A dysfunction is impairment in a form of hippocampal synaptic plasticity, producing deficits in short-term habituation and thence elevated and dysregulated levels of attention, a phenotype of relevance to schizophrenia and its cognitive aspects.

The excitatory neurotransmitter glutamate is considered second only to dopamine in the neurochemistry of schizophrenia. Interest can be traced back over fifty years [1] and especially to a series of post mortem brain studies in the late 1980s and early 1990s which reported alterations in glutamate receptor expression [2–9] and glutamate metabolism [10], and to papers which described relevant behavioural and cognitive effects of glutamatergic ligands [11, 12]. Interest has continued unabated since then, accompanied by various glutamatergic theories of schizophrenia, most of which invoke a central role for ‘hypofunction’ of the N-methyl-D-aspartate (NMDA) subtype of glutamate receptor, often linked to the neurodevelopmental origins of the disorder [13–18]. However, the exact nature of the proposed NMDA receptor hypofunction, and its causes, correlates and consequences, remained elusive. Now, building upon this extensive but ultimately circumstantial evidence, new genomic findings show that the NMDA receptor is part of the genetic aetiology of the syndrome.

NMDA receptors are heteromeric ion channels, comprising two obligate NR1 (also called GluN1) subunits, encoded by *GRIN1*, and two NR2 (GluN2) subunits (NR2A – NR2D, encoded by *GRIN2A*-*GRIN2D*); they may also include NR3 (GluN3) subunits (encoded by *GRIN3A* and *GRIN3B*). For review, see refs [19, 20]. The NR2 subunits contain the glutamate binding site and, via their C-terminal domain (CTD), interact with multiple intracellular proteins such as Ca^2+^/calmodulin-dependent protein kinase II (CamKII), post-synaptic density protein-95 (PSD-95), and Homer. Within the NR2 family, much attention has focused on NR2A and NR2B, since these comprise the predominant NR2 subunits in the forebrain [21]. Key differences between NR2A and NR2B include: (1) a developmental switch from NR2B to NR2A across human brain development [22, 23]; (2) a preferential synaptic as opposed to extra-synaptic localisation for NR2A; (3) their channel gating properties, with NR2A-containing receptors having larger current densities, higher open probability, lower agonist sensitivity, and more rapid recovery from desensitisation; (4) differing protein interactions and receptor trafficking via the CTD [24, 25]. Complementing these functional differences, NR2A and NR2B are involved differentially in learning and memory, synaptic plasticity, and pathophysiologies [24, 26]. Loss-of-function mutations in *GRIN2A* are known to cause seizure disorders, disorders of intellectual development, and speech and language disorders [27–30]; in the largest series of *GRIN2A* mutations reported to date (*n* = 284 people), 24% had a documented neuropsychiatric comorbidity, including two cases with schizophrenia [28].

A direct relevance of these findings to schizophrenia has now emerged from findings that both rare and common variants of *GRIN2A* are associated with genetic risk for the disorder. Here we review this evidence and revisit the findings from *Grin2a* mouse models to explore the likely phenotypic and mechanistic correlates of this genetic variation.

## *GRIN2A* and schizophrenia

Back-to-back papers in *Nature* last year provide a step-change in the evidence for involvement of the NMDA receptor in schizophrenia and, in particular, for a genetic contribution from *GRIN2A*.

**Fig. 1:**
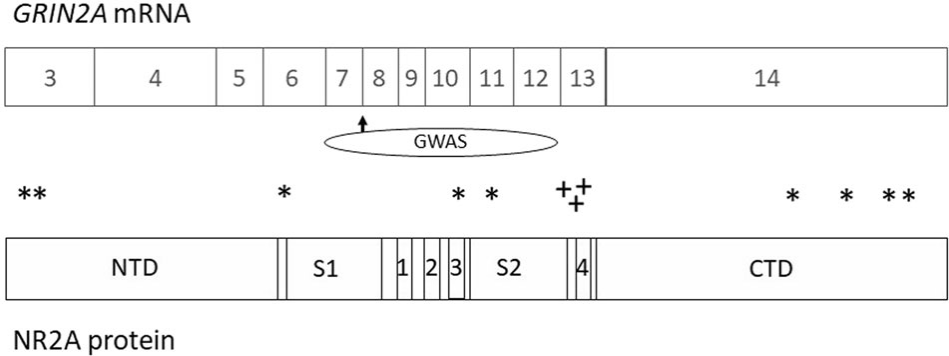
The coding exons (exons 3–14) of *GRIN2A* are shown, with location of the main functional domains of the encoded *NR2A* protein shown below. CTD C-terminal domain, 1–4 M1–M4 membrane domains, NTD N-terminal domain, S1 and S2 ligand-binding domains. *location of the protein-truncating rare variants associated with schizophrenia, +location of the probable damaging (MPC > 3) missense variants associated with schizophrenia. MPC (‘Missense badness, PolyPhen-2, Constraint’) score is a missense deleteriousness metric [72]. An MPC cut-off of 3 is stringent and implies a high likelihood that the variant is deleterious; at a lower threshold (MPC > 2), there was no excess of *GRIN2A* missense variants in schizophrenia cases [31]. Two controls had protein-truncating variants in the CTD (not shown). The approximate location of the GWAS-associated *GRIN2A* locus is shown as an ellipse, with the arrow denoting the index SNP rs9926049.

Singh and colleagues [31] meta-analysed exome sequencing data from 24,248 individuals diagnosed with schizophrenia and 97,322 individuals with no known psychiatric disorder. Using a stringent analytic approach, they identified ultra-rare variants within ten genes as conferring substantial risk for schizophrenia. *GRIN2A* was one of the genes, with nine protein-truncating mutations and three deleterious (MPC > 3) missense mutations identified in cases, compared to two and zero respectively in controls (*P* = 7.18 × 10^–6^; overall odds ratio 24.1 [95% CI 5.36–221]; see Fig. 1).

There are several interesting features of the *GRIN2A* rare variants reported in schizophrenia. Firstly, the majority are protein-truncating variants rather than missense mutations. As such the phenotypic consequences are most likely the result of haploinsufficiency and thence loss of function [32]. Loss of *GRIN2A* might result in compensatory *GRIN2B* over-expression, which would actually lead to an effective gain of function, given the characteristics of NMDA receptors containing each subunit. However, data from *Grin2a*^*+/−*^ and *Grin2a*^*−/−*^ rats show convincingly that *Grin2b* does not compensate, and that both knockouts and heterozygotes show reduced NMDA-evoked current density and more complete inhibition by the NR2B antagonist ifenprodil [28].

The functional correlates of the missense variants found in schizophrenia are unknown, but other *GRIN2A* point mutations can produce gain or loss of function, or other effects on receptor characteristics, depending in part on their domain location within the protein [27, 28, 33–35]. Notably, it is missense mutations rather than protein-truncating variants in *GRIN2A* that are associated with risk of severe developmental disorder and intellectual disabilities [31]. This suggests that the ‘noise’ introduced by a missense mutation leads to more extreme phenotypic effects than the ‘reduced signal’ that arises from haploinsufficiency. This relationship might also apply within schizophrenia, whereby cases associated with *GRIN2A* point mutations have more prominent cognitive impairments (and other neurodevelopmental comorbidities) than cases attributed to *GRIN2A* protein-truncating variants. This is currently unknown.

A further notable feature is that four of the schizophrenia-associated variants in *GRIN2A* occur in the CTD, whereas none occurred in this region in the 284 *GRIN2A*-associated neurodevelopmental disorder cases reported by Strehlow and colleagues [28]. On the one hand, this difference in location may contribute to the different phenotypes observed, and it also draws attention to the functions of the CTD [36–38] and to mouse models in which this domain is deleted (see below). On the other hand, Strehlow et al [28] also note the much greater tolerance to CTD variation and as such a variant in that region may be considered a priori less likely to be pathogenic [39].

In the companion paper, Trubetskoy et al. [40] conducted the largest genome-wide association study yet reported for schizophrenia, with up to 76,755 cases compared with up to 243,649 controls. *GRIN2A* was one of the 287 significant genomic loci, with the index single nucleotide polymorphism (SNP), rs9926049, being in the seventh intron and having an odds ratio of 1.057, *P* = 1.57 × 10^–10^ (Fig. 1). There is preliminary evidence that the risk genotype is associated with increased methylation, reduced mRNA expression, and altered splicing [41], together consistent with an overall hypofunctional effect, as for the rare variants. The strong evidence notwithstanding, it is of note that summary Mendelian randomisation did not provide positive evidence supporting *GRIN2A* as the causal gene within the locus [40]. Further investigations of the mechanism of association will thus need to confirm a causal role of common variation within *GRIN2A*, identify the genotype-related alterations in expression and whether it impacts on NR2A abundance, and investigate the possibility that the effects could be temporally specific (e.g. occurring at particular developmental stages) or spatially restricted (e.g. to some cell types, as discussed below).

## *Grin2a* mouse models

Prior to these new genetic findings in schizophrenia, *Grin2a* mouse models had already been well characterised, both electrophysiologically and behaviourally, reflecting the broad neuroscientific interest in NMDA receptors and their roles in neural functioning and synaptic plasticity. Though the phenotype of *Grin2a*^*−/−*^ mice is not entirely consistent between studies [42], the mice are clearly impaired in some facets of memory, exhibit reduced long term potentiation (LTP), and show altered electrophysiological properties of NMDA receptors, including faster decay rates [43–48].

Detailed behavioural analysis of *Grin2a*^*−/−*^ mice reveals a specific deficit in hippocampus-dependent short-term memory which results in a failure to habituate [47], and leading to locomotor hyperactivity when placed in a novel environment (DMB, unpublished observations). In marked contrast, long-term memory performance (on tasks like the Morris water maze and the spatial reference memory version of the radial maze) is not impaired. Notably, this phenotype is very similar to that seen in *Gria1*^−/−^ (GluA1, GluR1, GluR-A) AMPA glutamate receptor subunit knockout mice, which also exhibit a selective deficit in short-term habituation (with normal or even enhanced long-term memory), leading to elevated and unregulated levels of attention, locomotor hyperactivity and a hyperdopaminergic phenotype [49–52]. The similarity in these behavioural profiles potentially reflects a common mechanistic pathway whereby a specific form of hippocampal LTP is induced through activation of NR2A-containing NMDA receptors and subsequently expressed through the rapid trafficking of GluA1-containing AMPA receptors into the post-synaptic density [53].

Against this backdrop, the phenotype of *Grin2a*^*+/−*^ heterozygotes has also been examined recently, and is arguably of more relevance to schizophrenia than are the *Grin2a*^*+/−*^ knockouts, given the protein-truncating hemizygous *GRIN2A* mutations and thence the presumed haploinsufficiency. Herzog and colleagues [54] used EEG to show that *Grin2a*^*+/−*^ mice exhibit attenuated auditory steady-state responses at gamma frequencies, and increased gamma power during sleep, both features that have been observed in schizophrenia. Locomotor activity was also increased. A subsequent study reported a detailed transcriptomic and proteomic assessment and showed that *Grin2a* heterozygosity is associated with significant, diverse, and complex changes [55]. Strikingly, many of the findings are consistent with known or suspected differences in schizophrenia, including upregulation of markers of striatal dopamine signalling, reciprocal changes in prefrontal cortex and hippocampus, and down-regulation of glutamatergic synaptic markers. Moreover, schizophrenia-associated risk genes as identified by Trubetskoy et al. [40]. and Singh et al. [31] were enriched amongst the differentially expressed transcripts. Finally, it was notable that in most respects, *Grin2a*^*+/−*^ heterozygote mice showed changes that were similar in magnitude to *Grin2a*^*−/−*^ knockouts. Taken together, these findings provide a detailed picture of the molecular landscape associated with reduction or loss of *Grin2a*, and a convincing convergence with the pathophysiology of schizophrenia.

As noted earlier, several of the schizophrenia-associated variants in *GRIN2A* are located in the CTD (Fig. 1). As such, the phenotype of mice in which the CTD is selectively deleted (*Grin2a*^*ΔC/ΔC*^) is also of relevance [47, 56, 57]. Notably, their behavioural profile is similar to that of the *Grin2a* knockout even though the CTD mutants are able to form functional glutamate-gated cation channels [56]. In particular, *Grin2a*^*ΔC/ΔC*^ mice, like *Grin2a*^*−/−*^ knockouts, exhibit a selective deficit in hippocampus-dependent short-term memory but are capable of forming normal long-term memories [47]. This similarity supports the interpretation that the phenotype of both types of *Grin2a* mutant arise from the CTD, by impairing its physical interaction with intracellular proteins and thence the ability to transduce the receptor-mediated calcium ion influx into downstream effects. This convergence might imply a similar common pathway arising from the various forms of *GRIN2A* genetic involvement in schizophrenia.

Although mouse models with point mutations in *Grin2a* have not been extensively studied, data are consistent with the evidence from human studies that the phenotype is more severe than in knockouts. For example, Bertocchi et al [58] showed that the *Grin2a*^*N615S*^ mutant has audiogenic seizures, reduced hippocampal activity, attentional abnormalities and impaired associative learning.

A final issue to which the mouse models can contribute concerns the cell types within which the effects of *GRIN2A* variation are exerted. It is known that NR2A plays a key role in the function and maintenance of parvalbumin interneurons [59], a cell population often implicated in schizophrenia. Speculatively, a reduction of NR2A subunit-containing NMDA receptors could result in a decreased excitability of these cells, thereby disinhibiting pyramidal neurons and contributing to the putative excitation/inhibition imbalance of schizophrenia [60, 61]. In one prominent model of this kind, Lisman and colleagues [62] suggested that NMDA receptor hypofunction on parvalbumin interneurons, leading to disinhibition of hippocampal pyramidal cells might, in turn, drive an increase in activity of dopaminergic neurons in the ventral tegmental area. However, the variable behavioural phenotype in mice lacking NMDA receptors on parvalbumin interneurons [63–66], and the observation that responses to the NMDA receptor antagonist MK-801 are enhanced rather than reduced in these mice [66], indicates that additional studies are needed to localise the cells and circuits within which *GRIN2A* genetic variation contributes to schizophrenia-relevant phenotypes – including the deficits in short-term habituation noted earlier - and whether this occurs primarily by affecting the pharmacological characteristics of NMDA receptors themselves, or via downstream effects on synaptic plasticity.

## Future directions

Two recent high-profile papers [31, 40] put the glutamate hypothesis of schizophrenia onto a stronger genetic footing and centre it around *GRIN2A*. Inevitably, however, much remains to be discovered.

**Fig. 2:**
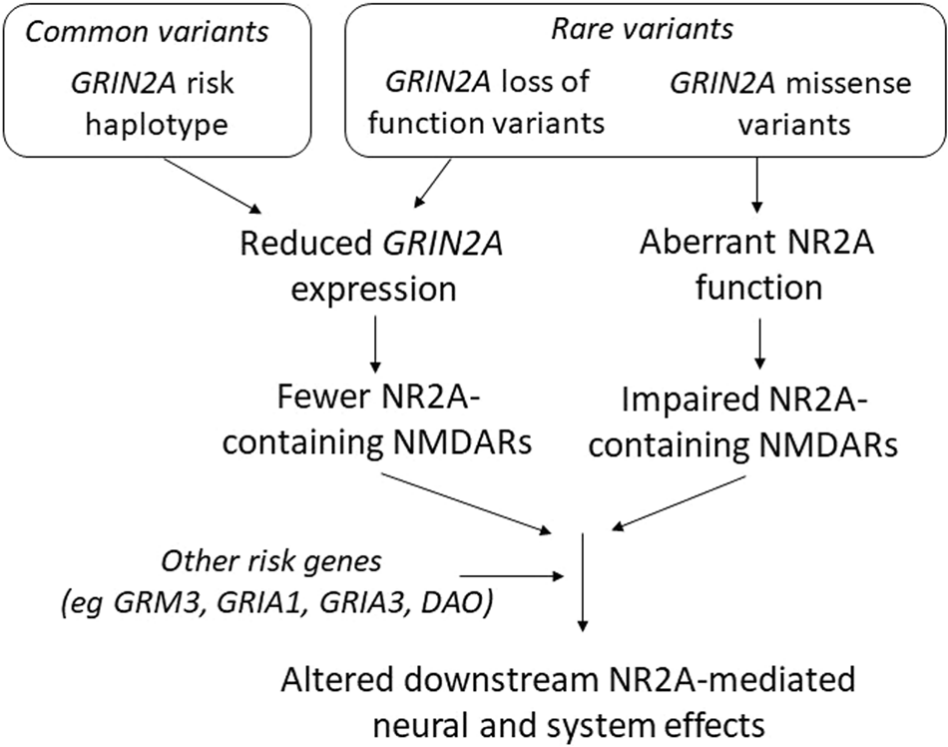
Simple and speculative schematic for the functional consequences of *GRIN2A* variants involved in schizophrenia. Common variants, and *GRIN2A* loss of function (protein truncating) variants, are proposed to lead to reduced mRNA expression and thence lower NR2A subunit abundance, whereas *GRIN2A* missense variants result in NR2A subunits with aberrant electrophysiological properties. These NR2A-mediated impairments in NMDA receptor function interact with other glutamatergic risk genes and the many other pathways and processes involved in schizophrenia pathophysiology.

Firstly, what do the schizophrenia-associated *GRIN2A* mutations and SNPs do to NR2A function? Is there a convergence (and, if so, what is it?) or are there multiple downstream effects? This question applies not only to the composition, distribution, and electrophysiological characteristics of the NMDA receptors themselves, but also to the broader intracellular and systems effects in which NMDA receptor signalling participates, such as synaptic plasticity (Fig. 2). The impact of *GRIN2A* variation on the interaction between NMDAR signalling and the dopamine system is also of particular interest.

Secondly, clarification is required as to the schizophrenia-related phenotypes to which *GRIN2A* genetic variation contributes, given the prior evidence that mutations in the gene primarily cause intellectual disability, epilepsy, and speech and language disorders. Whether the schizophrenia cases reported by Singh and colleagues [31] also had features of this kind is not clear, and more detailed genotype-phenotype characterisation will be critical in the future.

Thirdly, *GRIN2A* is not the only glutamatergic gene which is now strongly implicated in schizophrenia by rare and/or common variants. There is genome-wide association to loci for the AMPA receptor subunits *GRIA1* (see above) and *GRIA3*, the group II metabotropic glutamate receptor *GRM3*, and *DAO* (D-amino oxidase), the enzyme which metabolises the NMDA receptor co-agonist D-serine. There are also rare variants in *SP4*, a transcription factor which regulates *GRIN2A* expression. Each of these genes has been discussed with regard to its own involvement in schizophrenia [17, 50, 67–69] but the time is ripe for a more integrated analysis of how they contribute collectively to its glutamatergic pathophysiology (or pathophysiologies) and to the postulated aberrant synaptic plasticity.

Finally, the recent genomic findings encourage investigation of the therapeutic potential of selective NR2A-targetting drugs (such as NR2A positive allosteric modulators) for schizophrenia, as part of the broader interest in glutamatergic treatments for the disorder [18, 29, 70, 71].
